# A Game-Theoretical Winner and Loser Model of Dominance Hierarchy Formation

**DOI:** 10.1007/s11538-016-0186-9

**Published:** 2016-06-24

**Authors:** Klodeta Kura, Mark Broom, Anne Kandler

**Affiliations:** Department of Mathematics, City University London, Northampton Square, London, EC1V 0HB UK

**Keywords:** Resource holding potential, Dominance hierarchy, Expected payoff, Stopping time, Evolutionary game theory

## Abstract

Many animals spend large parts of their lives in groups. Within such groups, they need to find efficient ways of dividing available resources between them. This is often achieved by means of a dominance hierarchy, which in its most extreme linear form allocates a strict priority order to the individuals. Once a hierarchy is formed, it is often stable over long periods, but the formation of hierarchies among individuals with little or no knowledge of each other can involve aggressive contests. The outcome of such contests can have significant effects on later contests, with previous winners more likely to win (winner effects) and previous losers more likely to lose (loser effects). This scenario has been modelled by a number of authors, in particular by Dugatkin. In his model, individuals engage in aggressive contests if the assessment of their fighting ability relative to their opponent is above a threshold $\theta$. Here we present a model where each individual can choose its own value $\theta$. This enables us to address questions such as how aggressive should individuals be in order to take up one of the first places in the hierarchy? We find that a unique strategy evolves, as opposed to a mixture of strategies. Thus, in any scenario there exists a unique best level of aggression, and individuals should not switch between strategies. We find that for optimal strategy choice, the hierarchy forms quickly, after which there are no mutually aggressive contests.

## Introduction

Very often, animals that share the same territory engage in pairwise aggressive interactions leading to the formation of dominance hierarchies Hand (1986). Here we are interested in groups of animals that are meeting for the first time and have to engage in these aggressive interactions in order to divide their resources. In smaller groups of animals, the hierarchy tends to be linear where one individuals dominates all of the others, a second individual dominates all of the others in the group except the top-ranked individual and so on (see Addison and Simmel 1970; Barkan and Strahl 1986; Goessmann et al. 2000; Wilson 1971). In larger groups of animals, the hierarchy is more complex, where the position of especially lower-ranked individuals may be unclear, e.g. in chimpanzees, baboons, hyenas (Kummer 1984; Möller et al. 2006, 2001; Widdig et al. 2001). Some animals are more aggressive than others, and the level of aggressiveness depends upon many factors such as experience, the value of winning the contest and resource holding potential (RHP) (see e.g. Blanchard et al. 1988; Blanchard and Blanchard 1977; Moss et al. 1994; Takahashi and Lore 1983; Taylor 1982).In our model, RHP is simply the ability of an individual to win an escalated contest (Parker 1974), abstracted away from any particular causal effect. In reality, there are a large number of elements that determine the RHP. Very broadly, these elements can be divided into physical attributes, such as size, age and physical strength (intrinsic factors), and psychological attributes, such as prior experience (extrinsic factors).

In more detail, there are a lot of results demonstrating a strong correlation between RHP and body size (Alexander 1961; Bridge et al. 2000; Lindström 1992). For example, it has been observed that larger animals are more aggressive towards smaller ones and that they have more chances of winning an encounter (Frey and Miller 1972; Knights 1987). However, other results show that such physical attributes are not the only important determinant of RHP. For example, Brown et al. (2006) showed that 37.5 % of the group in house crickets won aggressive interactions, even though they had smaller body size. In Hofmann and Schildberger (2001), bigger individuals lost 30 % of the aggressive interactions.

Prior experience as well can have an important effect on the RHP of an individual. For example, if an individual has won more fights than it has lost in the past, it may increase its potential to win in the future.

The aim of this paper is to explore the relationship between extrinsic factors, in particular prior experience, and hierarchy formation. Therefore, we assume in our model that all individuals have identical physical abilities, so that the outcome of an encounter is significantly determined by past experience (although our results depend upon only a mechanical updating of RHP after a contest, so it would allow for real physical as well as psychological changes, too). In particular, we consider so-called winner and loser effects. The winner effect occurs when winning a previous contest increases the chances that an animal wins a subsequent contest. The loser effect occurs when a previous loss similarly increases the chances of defeat in the next contest.

A number of authors have analysed the influence of winner and loser effects on dominance hierarchy formation (e.g. Bonabeau et al. 1999; Dugatkin 1997; Dugatkin and Dugatkin 2007; Hemelrijk 2000). The first models were developed by Landau (Landau 1951a, b). He demonstrated the importance of the winner and loser effects: only when extrinsic factors were considered in addition to intrinsic ones, did the resulting hierarchies resemble those found in nature. Landau considered populations where winner and loser effects were operating together, but there is evidence that some groups of animals experience either winner or loser effects only (Bakker et al. 1989; Bergman et al. 2003; Lindquist and Chase 2009; Schuett 1997). Dugatkin and Dugatkin (Dugatkin 1997; Dugatkin and Dugatkin 2007) developed a model where these effects were considered in isolation, or both to be present in a group of 4 individuals. In Dugatkin (1997), each individual could only knew its own RHP after a win or loss, but they did not have any information about their opponents strength, except at time $t=1$. He predicted that when only the winner effect is at play, the emerging dominance hierarchies are linear and the strength of the winner effect is not important. Contrary, when only the loser effect is present, hierarchies where only the top-ranked individual is determined are found (the positions of the rest of the group stay unclear). When both winner and loser effects are present, nonlinear hierarchies emerged where only the first place, and sometimes the second place, was clear in the group. In Dugatkin and Dugatkin (2007), each individual was aware of their own RHP and they could make an imperfect estimate about their opponent’s RHP at each point in time. He concluded that overestimating or underestimating the opponent’s strength does not have any influence on linearity: in both cases, linear dominance hierarchies were established.

In Kura et al. (2015), we analysed the temporal dynamic and the average behaviour of dominance hierarchy formation for different combinations of winner and loser effects, using the model developed by Dugatkin (1997). We concluded that it is not necessary for a group of individuals to have perfect knowledge of each other’s RHP in order to establish a linear dominance hierarchy; only a little information about the current RHP estimation of an individual’s opponent is enough to establish a linear dominance hierarchy. We used different statistical measures such as the overlap between the distribution of the RHP of each individual over time to check for distinguishability between a pair of individuals. The index of linearity was used to measures how far from linearity each hierarchy is. Furthermore, we considered the question of how many fights are needed for a dominance hierarchy to be established, and we found that this number is relatively low.

In Dugatkin (1997) and Dugatkin and Dugatkin (2007) (as well as Kura et al. 2015), each individual had the same fixed level of aggression; they would retreat for the same excess of the number of wins over the number of losses. In this paper, we introduce game-theoretical elements in the form of aggressiveness level into this model. We assume that each individual can choose its own strategy, independent of their opponent’s strategy. We are particularly interested in determining the appropriate level of the aggression threshold and exploring whether a unique strategy, or mixture of strategies, emerges in the population considered. Our model set-up allows us to answer questions such as under what circumstances should an individual fight more in order to establish a higher rank in the hierarchy and when should it retreat? We use a framework similar to the Hawk–Dove model Maynard Smith (1982), where an individual can choose to either fight or concede, with each individual making its choice simultaneously. When two individuals choose to fight, they engage in an aggressive interaction; the winner will increase its RHP by a factor $1+V_{1}$, and the loser will reduce its RHP by a factor $1-C_{1}$. When one individual fights and the other concedes, the individual that chooses to fight increases its RHP by a factor $1+V_{2}$ and the retreating individual has its RHP reduced by a factor $1-C_{2}$. In the case when both individuals retreat, they have their RHP multiplied by $1-C_{2}$. Individuals choose their own strategies, meaning whether to fight and or to concede in an aggressive interaction given their history of fights won and lost, from a range of possible strategies. For each of these possible strategies, we will determine the resulting expected payoff and conclude whether the chosen strategy is beneficial to the individual or not. We will analyse two cases: when each individuals choose a strategy that enables them to fight in all interactions, and when they choose strategies that enable them to fight until a certain point in time (based upon how many contests they have won or lost) and retreat afterwards. We will determine the evolutionarily stable strategies (ESSs) for this fighting game, where an ESS is a strategy that when played by almost all members of the population cannot be invaded by any other strategy. We will also calculate the possible stopping times of the game for different strategies and analyse the relationship between the stopping time and the difference of the number of wins and losses for an individual.

As explained above, individuals fight for more access to resources and we will investigate the effects of different payoff functions on the ESSs within our model. In particular, we compare payoffs which depend upon the level of resource an individual receives to those which depend upon the proportion of the overall resource that it receives. The latter payoff function is particularly appropriate when resources are scarce. Once the dominance hierarchy is established, it is easier for the group to divide resources between them: the higher the position in the hierarchy, the higher the payoff. The division of resources has been analysed by different authors (see e.g. Broom and Ruxton 2001; Keller and Reeve 1994). We will use the concept of *reproductive skew* (Broom et al. 2009; Keller and Reeve 1994; Reeve and Keller 2001; Shen and Reeve 2010; Vehrencamp 1983), which refers to the distribution of reproductive rights in a group of animals. We will use the term more generally to refer to how limited resources, and hence, payoffs (which are generally proportional to reproductive levels in evolutionary games) are divided among our group. When the reproductive skew is high, the division of resources is uneven with the high-ranking individuals obtaining more resources than the lower-ranking ones (for example, see Drews 1993; Monnin and Ratnieks 1999; Rood 1980). In contrast, if the reproductive skew is low, the division of resources is even and all ranks of individuals have similar resource levels (see Brown 2014; Mangold et al. 2015). Further, we will explore the interplay between all three game-theoretical elements, $V_{i}$, $C_{i}$ and strategies $\theta _{x}$, and analyse whether there is a general pattern for the ESS when the $V_{i}$ and $C_{i}$ are increased (or decreased). Additionally, we develop a simulation framework to investigate the effect of the group size on the level of aggression. We note that Andersen et al. (2004) developed an alternative optimisation-based model to analyse the effect of group size on aggression level and showed that the theoretical results obtained are supported by experimental data observed in domesticated pigs; we discuss this in Sect. 6. Lastly, we compare our theoretical results with experimental evidence which is rather different for different groups of animals such as birds, farmed animals or fish (see e.g. Andersen et al. 2004; Bilčık and Keeling 2000; Estévez et al. 1997; Estevez et al. 2007; Kotrschal et al. 1993; Nicol et al. 1999; Syarifuddin and Kramer 1996; Turner et al. 2001).

## The Model

We assume a large population of social individuals living together in groups. At the beginning of the consideration, groups of size *N* are randomly formed, so that all individuals are members of a group and we analyse a specific group of *N* individuals. Each individual has an RHP value, which, as mentioned in the Introduction, is a measure of its ability to win an aggressive interaction (cf. Dugatkin 1997; Dugatkin and Dugatkin 2007) and which is altered by the outcome of each interaction. At the beginning, all individuals are assigned the same initial RHP, denoted by ${\hbox {RHP}}_{\hbox {initial}}$. We assume that all individuals know their own RHP and that of any opponent. In each round *t* ($t=1,..., T$), two individuals are randomly chosen to engage in an aggressive interaction, while the rest of individuals do not engage in any aggressive interactions. Through time, an individual’s RHP changes due to winning or losing (in reality, it will be mainly the extrinsic factors than change, but our model could cope with other eventualities equally well), while a win increases the RHP, a loss decreases it and each individual keeps track of the changes in their own RHP and that of its opponents. More precisely, suppose that at time *t* the two individuals pitted against each other are *x* and *y*. We denote by ${\hbox {RHP}}_{x,t}$ individual *x*’s RHP at time *t*. Individual *x* can decide to be aggressive or retreat once it has been chosen and this decision is based on the strategy $\theta _{x} \ge 0$ which is its aggression threshold.

Individual *x* fights individual *y* at this time (plays Hawk) if

1 $$\begin{aligned} \frac{{\hbox {RHP}}_{x,t}}{{\hbox {RHP}}_{y,t}}\ge \theta _{x} \end{aligned}$$

holds, otherwise it will retreat (play Dove), where ${\hbox {RHP}}_{y,t}$ and $\theta _{y}$ are the individual’s *y* RHP assessment score at time *t* and its aggression threshold, respectively. From the pairwise interaction, we get one of the following outcomes:

1. Both individuals *x* and *y* decide to engage in an aggressive interaction and the probability that *x* wins is given by
  2 $$\begin{aligned} P_{x,y}(t)=\frac{{\hbox {RHP}}_{x,t}}{{\hbox {RHP}}_{x,t}+{\hbox {RHP}}_{y,t}}, \end{aligned}$$
  and consequently, individual *y* wins with a probability $P_{y,x}(t)=1-P_{x,y}(t)$.
2. One individual engages in the aggressive interaction and the other retreats.
3. Both individuals decide not to fight (which is known as a double kowtow).

After a win, the RHP increases, and after a loss, it decreases. More precisely, if individual *x* wins and individual *y* loses, then they increase and decrease, respectively, their own RHP as follows:

3 $$\begin{aligned} {\hbox {RHP}}_{x,t+1}=(1+V_{1}){\hbox {RHP}}_{x,t}, \end{aligned}$$

4 $$\begin{aligned} {\hbox {RHP}}_{y,t+1}=(1-C_{1}){\hbox {RHP}}_{y,t}. \end{aligned}$$

If individual *x* wins and individual *y* retreats, then they increase and decrease, respectively, their own RHP as follows:

5 $$\begin{aligned} {\hbox {RHP}}_{x,t+1}=(1+V_{2}){\hbox {RHP}}_{x,t}, \end{aligned}$$

6 $$\begin{aligned} {\hbox {RHP}}_{y,t+1}=(1-C_{2}){\hbox {RHP}}_{y,t}. \end{aligned}$$

Equivalent changes to the RHPs apply if individual *y* wins.

If both individuals retreat (double kowtow), then they decrease their RHPs as follows:

7 $$\begin{aligned} {\hbox {RHP}}_{x,t+1}=(1-C_{2}){\hbox {RHP}}_{x,t}, \end{aligned}$$

8 $$\begin{aligned} {\hbox {RHP}}_{y,t+1}=(1-C_{2}){\hbox {RHP}}_{y,t}. \end{aligned}$$

In this model, $V_{1}$, $V_{2}$ are proportional increases in RHP and $C_{1}$, $C_{2}$ are proportional decrease in RHP where $V_{1}, V_{2} \ge 0$ and $C_{1}, C_{2} \in [0,1]$

The aim of each member of the population is to maximise its payoff at time *T*. In the following, we assume that the payoff function is defined as the natural logarithm of the RHP (which corresponds to the situation of unlimited resources) but consider in Sect. 3.5 the effects of an alternative payoff function (which corresponds to the situation of limited resources). Now there are two main reasons for considering the natural logarithm of the RHP. Firstly, while we want to keep to Dugatkin’s terminology as much as possible, the multiplicative nature of how the RHP increases means that RHP values can become large very quickly. If we would assume the expected RHP as the payoff, then even a minuscule chance of winning enough contests to be the top individual would be worth almost any risk. Considering the logarithm means that winning (losing) any contest increases (decreases) the payoff by the same amount irrespective of the current RHP, which seems reasonable. Secondly, taking the natural logarithm of the RHP guarantees that the payoffs increase in precisely the same way as in evolutionary matrix games, and in particular the Hawk–Dove game, which we use as an analogy in this paper.

This model set-up allows us to track the changes in RHP of all *N* individuals at the time points $t=1,\ldots ,T$ and therefore to evaluate which strategy $\theta$ results in the highest payoff over time. In this context, the ESS introduced by Maynard (1974) proves to be an important concept. An ESS is a strategy, that if adopted by a population, cannot be invaded by any other rare strategy. In general, we can have more than one ESS. In an *N*-player game, strategy $\theta _{x}$ is an ESS if either:

1. $$E[\theta _{x};\theta ^{N-1}_{x}]>E[\theta _{y};\theta ^{N-1}_{x}]$$ or
2. $$E[\theta _{x};\theta ^{N-1}_{x}]=E[\theta _{y};\theta ^{N-1}_{x}]$$ and $$E[\theta _{x},\theta ^{N-2}_{x},\theta _{y}]>E[\theta _{y},\theta ^{N-2}_{x},\theta _{y}]$$,

$\forall \theta _{y} \ne \theta _{x}$, where $E[\theta _{x};\theta ^{i}_{x},\theta ^{N-1-i}_{y}]$ is the expected payoff of an individual playing strategy $\theta _{x}$ against *i* individuals playing strategy $\theta _{x}$ and $N-i-1$ individuals playing strategy $\theta _{y}$, respectively Broom et al. (1997).

For Sect. 3, where we consider two-player games only, the ESS definition reduces to:

1. $$E[\theta _{x},\theta _{x}]>E[\theta _{y},\theta _{x}]$$ or
2. $$E[\theta _{x},\theta _{x}]=E[\theta _{y},\theta _{x}]$$ and $$E[\theta _{x},\theta _{y}]>E[\theta _{y},\theta _{y}]$$,

$\forall \theta _{y} \ne \theta _{x}$, where $E[\theta _{x},\theta _{x}]$ is the expected payoff of individual *x* against individual *y* with strategies $\theta _{x}$ and $\theta _{y}$, respectively.

**Fig. 1:**
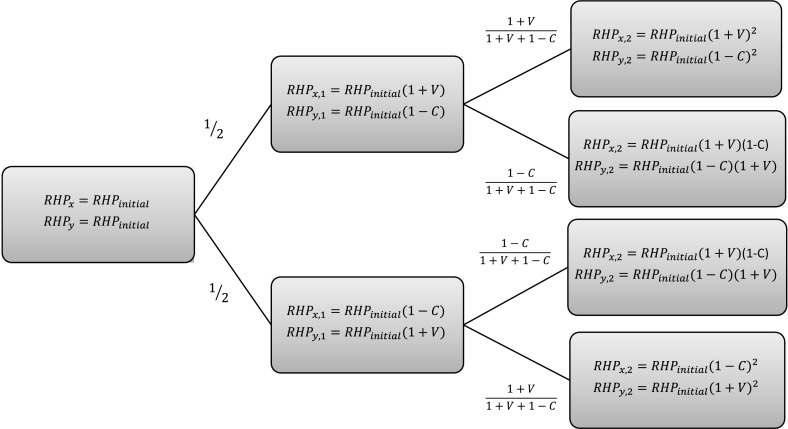
RHP of individual *x* and individual *y* at times $t=1$ and $t=2$ when they both start with the same ${\hbox {RHP}}_{\hbox {initial}}$ and always fight ($\theta _{x}=\theta _{y}=0$)

## The Two-Individual Model

For simplicity, in this section we consider groups of two individuals only. This will allow us to find some analytical results which will give us general insights into the dynamic of our model. We will then generalise to larger groups in Sect. 4.

### Expected Payoffs When Players Always Fight ($\theta _{x}=\theta _{y}=0$)

We assume that both individuals, denoted by *x* and *y*, possess the same ${\hbox {RHP}}_{\hbox {initial}}$ values. Further, individuals *x* and *y* play the strategies $\theta _{x}=\theta _y=0$, meaning that both individuals will fight until time *T* (cf. Eq. 1). In this section and throughout the paper, we assume $V_{1}=V_{2}=V$, $C_{1}=C$, $C_{2}=0$. This implies that winning a fight and having your opponent retreat has the same effect on the RHP. But contrary to Dugatkin (1997), we do not assume that losing a fight and retreating has the same effect on the RHP. This seems plausible as it is similar to the Hawk–Dove model to which we refer, in the sense that the loss of a fight is like an injury (whether a real injury or a psychological one). Figure 1 illustrates the possible RHP values of individual *x* at times $t=1$ and $t=2$. For example, the expected payoff of individual *x* at $t=1$, denoted by $E[\ln ({\hbox {RHP}}_{x,1})]$ is equal to

$$\begin{aligned} E[\ln ({\hbox {RHP}}_{x,1})]=\dfrac{1}{2}\ln ({\hbox {RHP}}_{{\hbox {initial}}}(1+V))+\dfrac{1}{2}\ln ({\hbox {RHP}}_{{\hbox {initial}}}(1-C)). \end{aligned}$$

An individual either wins or loses a fight, and we denote a win (loss) in the *k*th contest by $j_{k}=1$ ($j_{k}=0$). Thus, at time *t* individual *x* has $a_{t}$ wins and $b_{t}$ losses which are given as follows:

9 $$\begin{aligned} a_{t}=\sum _{k=1}^{t}j_{k} \end{aligned}$$

and

10 $$\begin{aligned} b_{t}=t-\sum _{k=1}^{t}j_{k}. \end{aligned}$$

The RHP for individual *x*, having won $a_{t}$ contests and lost $b_{t}$, will be denoted by $R_{a_{t},b_{t}}$ and is given by [cf. equations (3) and (4)]

$$\begin{aligned} R_{a_{t},b_{t}}{=}{\hbox {RHP}}_{{\hbox {initial}}}(1+V)^{a_{t}}(1-C)^{b_{t}}={\hbox {RHP}}_{{\hbox {initial}}}(1+V)^{\sum _{k=1}^{t}j_{k}}(1-C)^{t-\sum _{k=1}^{t}j_{k}}. \end{aligned}$$

The probability of winning after $a_{t}$ wins and $b_{t}$ losses at time *t* will be denoted by $W_{a_{t},b_{t}}$, whereas the probability of losing will be denoted by $L_{a_{t},b_{t}}=1-W_{a_{t},b_{t}}$. From equation (2), we obtain

$$\begin{aligned} W_{a_{t},b_{t}}=\frac{(1+V)^{a_{t}}(1-C)^{b_{t}}}{(1+V)^{a_{t}}(1-C)^{b_{t}}+(1+V)^{b_{t}}(1-C)^{a_{t}}}. \end{aligned}$$

If we consider all combinations of wins and losses and consider $\ln ({\hbox {RHP}})$, then the overall expected payoff is given by

11 $$\begin{aligned} E[\ln ({\hbox {RHP}}_{x,T})]=\sum _{j_{1}=0}^{1} \sum _{j_{2}=0}^{1}\sum _{j_{3}=0}^{1}....\sum _{j_{T}=0}^{1}\ln (R_{a_{T},b_{T}})\prod _{i=1}^{T}W^{j_{i}}_{a_{T},b_{T}}L^{1-j_{i}}_{a_{T},b_{T}}. \end{aligned}$$

where $a_{T}$ and $b_{T}$ are given by equations (9) and (10).

### Individuals with General Strategies $\theta _{x}$ and $\theta _{y}$

In this section, we analyse the expected payoffs for individuals *x* and *y* when they have potentially nonzero and different strategies $\theta _{x}$ and $\theta _{y}$, respectively. We start by deriving a general criterion for the number of losses necessary so that an individual retreats. Suppose that at time *t* individual *x* has won $a_{t}$ contests against individual *y* and lost $b_{t}$. Then, its RHP will be ${\hbox {RHP}}_{x,t}=R_{a_{t},b_{t}}$. In contrast, individual *y* has won $b_{t}$ contests and lost $a_{t}$ against individual *x* resulting in a RHP of $RHP_{y,t}=R_{b_{t},a_{t}}$. Thus, from equations (3)–(6) we obtain:

$$\begin{aligned} R_{a_{t},b_{t}}={\hbox {RHP}}_{{\hbox {initial}}}(1+V)^{a_{t}}(1-C)^{b_{t}} \end{aligned}$$

and

$$\begin{aligned} R_{b_{t},a_{t}}={\hbox {RHP}}_{{\hbox {initial}}}(1+V)^{b_{t}}(1-C)^{a_{t}}. \end{aligned}$$

The next interaction between the individuals *x* and *y* will result in a fight if equation (1) holds for both individuals. In other words, the following two equations have to be satisfied simultaneously

12 $$\begin{aligned} \frac{{\hbox {RHP}}_{x,t}}{{\hbox {RHP}}_{y,t}}= \frac{R_{a_{t},b_{t}}}{R_{b_{t},a_{t}}}=(1+V)^{a_{t}-b_{t}}(1-C)^{b_{t}-a_{t}}=\left( \frac{1+V}{1-C}\right) ^{a_{t}-b_{t}}\ge \theta _{x} \end{aligned}$$

and

13 $$\begin{aligned} \frac{{\hbox {RHP}}_{y,t}}{{\hbox {RHP}}_{x,t}}=\frac{R_{b_{t},a_{t}}}{R_{a_{t},b_{t}}}=(1+V)^{b_{t}-a_{t}}(1-C)^{a_{t}-b_{t}}=\left( \frac{1+V}{1-C}\right) ^{b_{t}-a_{t}}\ge \theta _{y}. \end{aligned}$$

Next, we take the logarithm of equations (12) and (13) on both sides and obtain

14 $$\begin{aligned} (a_{t}-b_{t})\ge \frac{\ln (\theta _{x})}{\ln (1+V)-\ln (1-C)} \end{aligned}$$

and

15 $$\begin{aligned} (b_{t}-a_{t})\ge \frac{\ln (\theta _{y})}{\ln (1+V)-\ln (1-C)}. \end{aligned}$$

We define

16 $$\begin{aligned} d_{x}=\frac{-\ln (\theta _{x})}{\ln (1+V)-\ln (1-C)} \end{aligned}$$

and

17 $$\begin{aligned} d_{y}=\frac{-\ln (\theta _{y})}{\ln (1+V)-\ln (1-C)} \end{aligned}$$

where $d_{x}$ and $d_{y}$ are both positive numbers for any pair of individuals which do not concede immediately. As equations (14) and (15) have to be fullfilled simultaneously, we obtain

18 $$\begin{aligned} -d_{x}\le a_{t}-b_{t} \le d_{y}. \end{aligned}$$

This means that if the excess of the number of wins over the number of losses is within $[-d_{x},d_{y}]$, individuals *x* and *y* will engage in a fight. If both individuals start by fighting and the first condition to not hold is $a_{t}-b_{t}\le d_{y}$, then we have a case where individual *y* decides to retreat and individual *x* to fight. After retreating for the first time, an individual then retreats in every contest until time *T*. Consequently, after *y* has retreated, individual *x* increases its RHP for every contest. By contrast, if the first condition to not hold is $-d_{x}\le a_{t}-b_{t}$, then individual *x* decides to retreat and individual *y* increases its RHP for every contest. The situation where both individuals retreat only occurs if this happens at $t=1$.

We define the time when individual *x* retreats by

19 $$\begin{aligned} T_{s}(x)=\text {min}\{t\ge 1: a_{t}-b_{t}<-d_{x}\}. \end{aligned}$$

$T_s(x)$ will be called the *x*-stopping time. The *y*-stopping time $T_{s}(y)$ is defined similarly. Clearly, in any contest exactly one of these values will be finite; the time of the last contest where both individuals fight is given by the stopping time $T_{s}$, where

20 $$\begin{aligned} T_{s}=\text {min}\{ T_{s}(x), T_{s}(y)\}. \end{aligned}$$

Then, the expected payoff $E[\ln ({\hbox {RHP}}_{x,T})]$ at time *T* is given by:

21 $$\begin{aligned} E[\ln ({\hbox {RHP}}_{x,T})]=\sum _{j_{1}=0}^{1} \sum _{j_{2}=0}^{1}....\sum _{j_{T_{s}}=0}^{1}\ln \left[ R_{a_{T},b_{T}}(1+V)^{(T-T_{s})I_{1}}\right] \prod _{i=1}^{T_{s}}W^{j_{i}}_{a_{T}, b_{T}}L^{1-j_{i}}_{a_{t}, b_{t}} \end{aligned}$$

where

$$\begin{aligned} I_{1} = {\left\{ \begin{array}{ll} 0 &{} \text {if } a_{t}-b_{t} < d_{x} \\ 1 &{} \text {if } a_{t}-b_{t} > d_{y} \end{array}\right. } \end{aligned}$$

and $(1+V)^{(T-T_{s})I_{1}}$ is the multiplicative increase in RHP that individual *x* gets after the stopping time $T_{s}$. It follows from inequality (18) and the fact that $a_{t}-b_{t}$ is an integer that all $\theta$ values within a certain interval result in the same expected payoff (for fixed *V* and *C*). We denote those intervals of strategy values by $[\theta _{x,{\hbox {min}}}, \theta _{x,{\hbox {sup}}})$ where $\theta _{x,{\hbox {sup}}}$ is the value of $\theta _{x}$ that corresponds to $\lfloor d_{x}\rfloor$ and $\theta _{x,{\hbox {min}}}$ the value of $\theta _{x}$ that corresponds to $\lceil d_{x} \rceil$. The intervals are closed at the lower bound and open at the upper bound and $\theta _{x,{\hbox {min}}}<\theta _{x,{\hbox {sup}}}$. We set

22 $$\begin{aligned} k'_{x}= \lfloor d_{x} \rfloor =\left\lfloor \frac{-\ln (\theta _{x,{\hbox {sup}}})}{\ln (1+V)-\ln (1-C)} \right\rfloor \end{aligned}$$

and obtain

$$\begin{aligned} \theta _{x,{\hbox {sup}}}=\left( \frac{1-C}{1+V}\right) ^{k'_{x}}. \end{aligned}$$

Further, we set $k_{x}=\lceil d_{x} \rceil$. The corresponding strategy value $\theta _{x}$ for $k_{x}$ is $\theta _{x,{\hbox {min}}}$ and we have

23 $$\begin{aligned} k_{x}=\lceil d_{x} \rceil =\left\lceil \frac{-\ln (\theta _{x,{\hbox {min}}})}{\ln (1+V)-\ln (1-C)}\right\rceil \end{aligned}$$

which results in

$$\begin{aligned} \theta _{x,{\hbox {min}}}=\left( \frac{1-C}{1+V}\right) ^{k_{x}}. \end{aligned}$$

Similarly to the above, for given *V* and *C* there is a range of $\theta$ values that correspond to a given *k*. Importantly, each strategy $\theta$ from that range results in the same payoff. We note, however, that this range changes for different *V* and *C*. For simplicity, we shall assume that individual *x* chooses the middle value from $[\theta _{x,{\hbox {min}}}, \theta _{x,{\hbox {sup}}})$, and this strategy will be denoted by $\theta _{x,{\hbox {rep}}}$ as the representative strategy of the $[\theta _{x,{\hbox {min}}}, \theta _{x,{\hbox {sup}}})$ range

24 $$\begin{aligned} \theta _{x,{\hbox {rep}}}=\left( \frac{1-C}{1+V}\right) ^{k_{x}}\left( \frac{2+V-C}{2(1-C)}\right) . \end{aligned}$$

### Stopping Time $T_{s}$

The expected payoff $E[\ln ({\hbox {RHP}}_{x,T})]$ given by equation (21) depends on the stopping time $T_s$. In this section, we explore the properties of $T_s$ as defined by equation (20), in particular its distribution.

To do so, we firstly determine the values of $k_{x}$ and $k_{y}$ for individuals *x* and *y* with strategies $\theta _{x}$ and $\theta _{y}$, respectively. The time when the random process $a_{t}-b_{t}$ is equal to $k_{x}$ or $k_{y}$ represents the stopping time. For instance, individual *x* would not engage in aggressive interactions when $a_{t}-b_{t}\le -k_{x}$ and the stopping time defined in equation 19 can be written alternatively as

25 $$\begin{aligned} T_{s}(x)={\hbox {min}}\{t\ge 1 : a_{t}-b_{t}\le -k_{x} \}. \end{aligned}$$

But which values can the stopping time $T_s(x)$ assume? The earliest possible *x*-stopping time is $T=k_{x}$, i.e. individual has $k_{x}$ consecutive wins from the start of the interaction. The next possible stopping time will be at $k_{x}+2$, where a single win by individual *x* within the first $k_{x}$ interactions has to be met by a total of $k_{x}+1$ wins by *y*. In general, the stopping times for individual *x* will be given by $k_{x}+(2n)_{n\ge 0}$. Consequently, the stopping times for individual *y* will be given by $k_{y}+(2n)_{n\ge 0}$. Thus, $T_{s}=\text {min}\{ T_{s}(x), T_{s}(y)\}$ can assume the following values

26 $$\begin{aligned} T_{s} = \left\{ \begin{array}{@{}l@{ }l} 1 &{}: {k}_{x}={k}_{y} = 1\\ {{\hbox {min}}}\{{k}_{x},{k}_{y}\} + (2n)_{n\ge 0} &{}: {k}_{x}+{k}_{y}\ \text {even}\\ {{\hbox {max}}}\{{k}_{x},{k}_{y}\} + {n} + \text {even numbers in}\ [{\hbox {min}}\{{k}_{x},{k}_{y}\}, {{\hbox {max}}}\{{k}_{x},{k}_{y}\}] &{}: {k}_{x}+{k}_{y}\ \text {odd}, \\ &{}\ \ {\hbox {min}}\{{k}_{x},{k}_{y}\}\ \text {odd} \\ {{\hbox {max}}}\{{k}_{x},{k}_{y}\} + {n} + \text {odd numbers in}\ [{\hbox {min}}\{{k}_{x},{k}_{y}\}, {{\hbox {max}}}\{{k}_{x},{k}_{y}\}] &{}: {k}_{x}+{k}_{y}\ \text {odd}, \\ &{} \ \ {\hbox {min}}\{{k}_{x},{k}_{y}\}\ \text {even} \end{array} \right. \nonumber \\ \end{aligned}$$

In summary, the stopping time defines the exact time when one individual starts to retreat for different strategy combinations. It also gives the number of possible interactions that need to be observed in order to distinguish between a pair of individuals, so that in our model the second individual will always concede to the first (for a different interpretation of this concept, see Kura et al. 2015).

Note that it is possible for our model to generate one experience, a winner effect or a loser effect, without the other. For example, for $V > 0$ and $C=0$ we have a case when only the winner effect is in place. Tables 5 and 6 show the expected payoffs for different strategic values when $V=0.1$ and $C=0$. On the other hand, when $C > 0$ and $V=0$, illustrated by Tables 7 and 8, we have a case when only the loser effect is operating.

In the next section, we derive the distribution of $T_s$ for the parameter constellation $V=C=0.1$ (both winner and loser effect are influencing RHP).

### Example: $V=C=0.1$

To illustrate the findings of the last sections, we consider an example by assuming the parameters $V=0.1$, $C=0.1$ and $T=20$. In particular, we calculate the expected payoffs $E[\ln ({\hbox {RHP}}_{x,20})]$ for different combinations of strategies $\theta _{x}$ and $\theta _{y}$, determine the unique ESS and derive the distribution of the stopping time $T_s$.In this section and throughout the paper, we will assume that ${\hbox {RHP}}_{{\hbox {initial}}}=10$.

Firstly, we determine the representative strategies to $k_{x}=1,2,3,4,5,6,7,8$ by using equation (24). Note that there is a range of strategies $\theta _{x}$ that correspond to the same value of $k_{x}$ and we take the middle one as described in Sect. 3.2. We obtain the following mappings (the same values apply for individual *y* as well).

$$k_{x}=1\Rightarrow \theta _{x,{\hbox {rep}}}=0.91$$, $$k_{x}=2\Rightarrow \theta _{x,{\hbox {rep}}}=0.74$$, $$k_{x}=3\Rightarrow \theta _{x,{\hbox {rep}}}=0.61$$, $$k_{x}=4\Rightarrow \theta _{x,{\hbox {rep}}}=0.50$$,

$$k_{x}=5\Rightarrow \theta _{x,{\hbox {rep}}}=0.41$$, $$k_{x}=6\Rightarrow \theta _{x,{\hbox {rep}}}=0.33$$, $$k_{x}=7\Rightarrow \theta _{x,{\hbox {rep}}}=0.27$$, $$k_{x}=8\Rightarrow \theta _{x,{\hbox {rep}}}=0.22$$.

For this set of strategies, we then calculate the expected payoffs $E[\ln ({\hbox {RHP}}_{x,20})]$ for individual *x* and $E[\ln ({\hbox {RHP}}_{y,20})]$ for individual *y* by using equation (21). Table 1 represent the matrix of payoffs for different combinations of strategies $\theta _{x}$ and $\theta _{y}$.

**Table 1:** The matrix of payoffs where each entry represent the expected payoff $E[\ln ({\hbox {RHP}}_{x,T})]$ at time $T=20$ [calculated by equation (21)] for different strategies $\theta _x$ and $\theta _y$

	$k_{y}=1$ ($\theta _{y}=0.91$)	$k_{y}=2$ ($\theta _{y}=0.74$)	$k_{y}=3$ ($\theta _{y}=0.61$)	$k_{y}=4$ ($\theta _{y}=0.50$)	$k_{y}=5$ ($\theta _{y}=0.41$)	$k_{y}=6$ ($\theta _{y}=0.33$)	$k_{y}=7$ ($\theta _{y}=0.27$)	$k_{y}=8$ ($\theta _{y}=0.22$)
$k_{x}=1$ ($\theta _{x}=0.91$)	3.2000	2.8700	2.7300	2.6500	2.6000	2.5800	2.5600	2.5400
$k_{x}=2$ ($\theta _{x}=0.74$)	3.4400	3.0600	2.8700	2.7600	2.7000	2.6600	2.6200	2.6000
$k_{x}=3$ ($\theta _{x}=0.61$)	3.5000	3.1000	2.8900	2.7700	2.6900	2.6500	2.6100	2.6000
$k_{x}=4$ ($\theta _{x}=0.50$)	3.5100	3.0800	2.8700	2.7400	2.6700	2.6200	2.5600	2.5600
$k_{x}=5$ ($\theta _{x}=0.41$)	3.5000	3.0500	2.8400	2.700	2.6200	2.5700	2.5400	2.5200
$k_{x}=6$ ($\theta _{x}=0.33$)	3.4600	3.0200	2.7900	2.6600	2.6000	2.5300	2.5000	2.4700
$k_{x}=7$ ($\theta _{x}=0.27$)	3.4300	2.9900	2.7700	2.6200	2.5600	2.5000	2.4800	2.4500
$k_{x}=8$ ($\theta _{x}=0.22$)	3.4200	2.9500	2.7300	2.6000	2.5100	2.4800	2.4400	2.4100

**Fig. 2:**
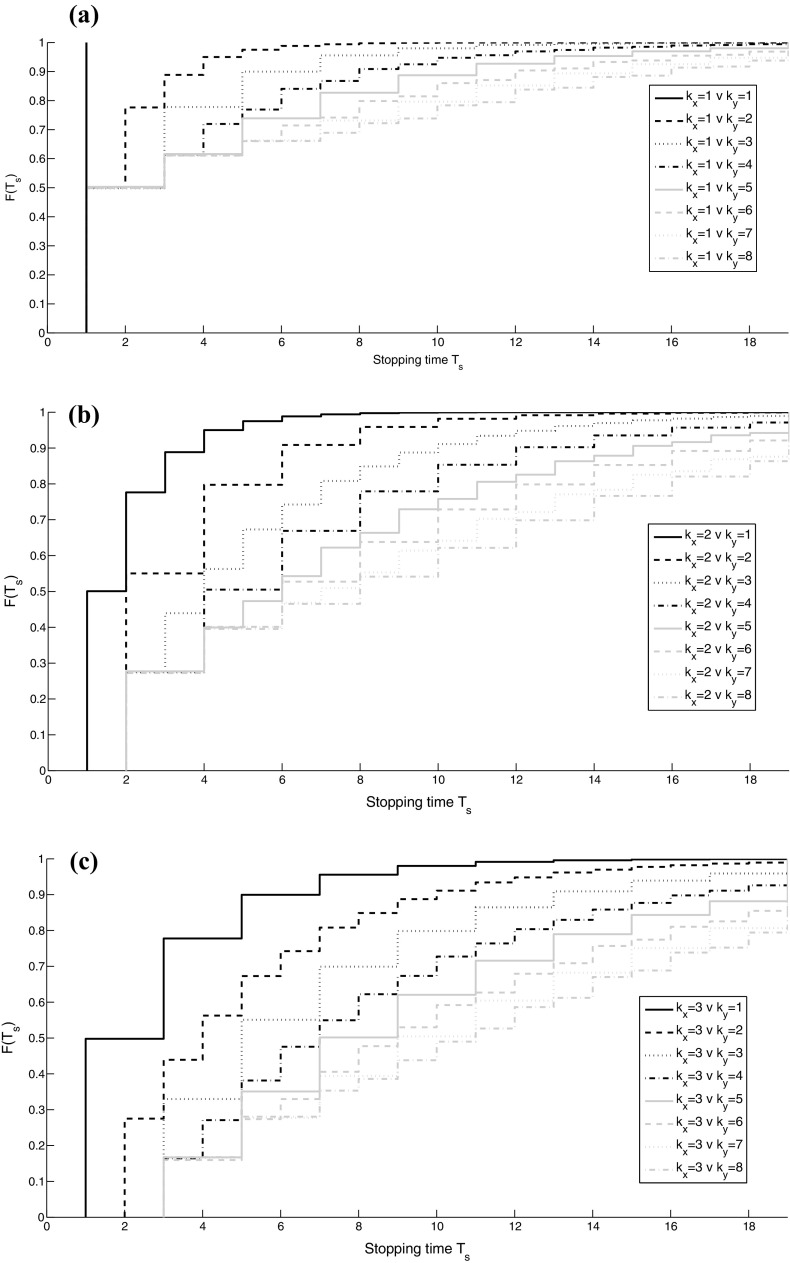
The distribution function of the stopping time for the case when $V=C=0.1$, $k_{y}=1,2,\ldots ,8$ and **a** $k_{x}=1$, **b** $k_{x}=2$, **c** $k_{x}=3$. Note that parts of the distribution functions are overlaid by other distribution functions, e.g. all lines in (**a**), include the segment with starting coordinate (1, 0) and ending coordinate $(1, \dfrac{1}{2})$

Now for each strategy, we can find the best response, i.e. for each column of Table 1 we find the highest payoff and use the “diagonal rule ”to find the ESS. The diagonal rule states that if any value on the diagonal of the matrix of payoffs is larger than all the values in the same column, then the corresponding pure strategy is an ESS. We note that for a pure ESS, all our results satisfy ESS condition 1 ; condition 2 is only achieved when mixtures are present, which we do not get in our example. In this example, we obtain $\theta =0.61$, corresponding to $k=3$, as the unique ESS. Note that there is a range of strategies $[\theta _{x,{\hbox {min}}}, \theta _{x,{\hbox {sup}}})=[0.55, 0.67]$ that corresponds to $k=3$. Thus, any strategy from this range results in the same expected payoff and is therefore equivalent to our ESS. Lastly, we derive the distribution of the stopping time $T_{s}$. For example, when $\theta _{x}=0.5$ (corresponding to $k_{x}=4$) and $\theta _{y}=0.7$ (corresponding to $k_{y}=2$), $T_s$ can only assume the values $(k_{y}+2n)_{n\ge 0}$ because $k_{x}+k_{y}=6$ is an even number [see equation (26)]. But how does this distribution change when $k_{x}$ and $k_{y}$ are varied? To explore this, we assume that individual *x* has a strategy $\theta _{x}$ corresponding to $k_{x}=1,2,3$ and his opponent has strategies $\theta _{y}$ corresponding to $k_{y}\in [1,8]$. We choose the value 8 as an upper bound for $k_{y}$ as an arbitrary large cut-off value which corresponds to small values of $\theta$, but we could have chosen any other high value. Figure 2 shows the distribution functions of the stopping time for various combinations of $k_{x}$ and $k_{y}$ for $V=C=0.1$.

Figures 2 illustrates that a pair of individuals will fight longer for higher values of $k_{x}$ and $k_{y}$. The reason behind this is that larger values of *k* correspond to smaller strategy values $\theta$, and hence, equation (1) implies that the individuals will fight longer. In this example, one of the individuals *x* and *y* has started retreating before time *T*, for most of the possible cases. This means that observing 20 interaction would allow us to distinguish between the two individuals almost with certainty. As we increase the values of $k_{x}$ and $k_{y}$, the probability of retreating before $T=20$ is decreased.

### An Alternative Payoff Function

Table 1 shows the expected payoff of individuals *x* and *y* after $T_{max}=20$ possible interactions using equation (21). In this section, we explore how limited resources are divided between the two individuals based on an alternative payoff function. We will use the concept of reproductive skew as discussed in Broom et al. (2009), Keller and Reeve (1994), Reeve and Keller (2001), Shen and Reeve (2010), Vehrencamp (1983). In this case, the expected payoff for individual *x* after 20 interactions is given by function:

27 $$\begin{aligned} E[\theta _{x},\theta _{y}]=E\left[ \frac{\ln ({\hbox {RHP}}_{x,20})}{\ln ({\hbox {RHP}}_{x,20})+\ln ({\hbox {RHP}}_{y,20})}\right] . \end{aligned}$$

Consequently, the expected payoff for individual *y* is given by function

$$\begin{aligned} E[\theta _{y},\theta _{x}]=E\left[ \frac{\ln ({\hbox {RHP}}_{y,20})}{\ln ({\hbox {RHP}}_{x,20})+\ln ({\hbox {RHP}}_{y,20})}\right] . \end{aligned}$$

The results are given in Table 2.

**Table 2:** Division of resources for different values of *k*, when $V=C=0.1$

	$k_{y}=1$ ($\theta _{y}=$1)	$k_{y}=2$ ($\theta _{y}=0.7$)	$k_{y}=3$ ($\theta _{y}=0.6$)	$k_{y}=4$ ($\theta _{y}=0.5$)	$k_{y}=5$ ($\theta _{y}=0.4$)	$k_{y}=6$ ($\theta _{y}=0.35$)	$k_{y}=7$ ($\theta _{y}=0.27$)	$k_{y}=8$ ($\theta _{y}=0.23$)
$k_{x}=1$ ($\theta _{x}=1$)	0.5000	0.4585	0.4417	0.4353	0.4344	0.4354	0.4360	0.4385
$k_{x}=2$ ($\theta _{x}=0.7$)	0.5415	0.5000	0.4825	0.4777	0.4765	0.4777	0.4806	0.4825
$k_{x}=3$ ($\theta _{x}=0.6$)	0.5583	0.5175	0.5000	0.4941	0.4928	0.4949	0.4973	0.4999
$k_{x}=4$ ($\theta _{x}=0.5$)	0.5647	0.5223	0.5059	0.5000	0.4992	0.5013	0.5034	0.5066
$k_{x}=5$ ($\theta _{x}=0.4$)	0.5656	0.5235	0.5072	0.5008	0.5000	0.5014	0.5046	0.5077
$k_{x}=6$ ($\theta _{x}=0.35$)	0.5646	0.5223	0.5051	0.4987	0.4986	0.5000	0.5026	0.5054
$k_{x}=7$ ($\theta _{x}=0.27$)	0.5640	0.5194	0.5027	0.4966	0.4954	0.4974	0.5000	0.5033
$k_{x}=8$ ($\theta _{x}=0.23$)	0.5615	0.5175	0.5001	0.4934	0.4923	0.4946	0.4967	0.5000

From Table 2, we find that $\theta =0.4$ (corresponding to $k=5$) is the ESS. Comparing this result with the result obtained from Table 1, we notice that they differ; when using this alternative payoff function, we obtain $k=5$ as the ESS, while for the original payoff function used in Sect. 3.4, the ESS is $k=3$. This differences are related to the amount of the available resources, in particular whether they are plentiful or limited. We assume that for plentiful resources, the absolute RHP is more important, but for scarce resources shared between group members, the relative RHP is the key element. If an individual needs to maximise the RHP, then it should fight less compared to the situation where it needs to maximise the division of limited resources. In this latter case, the individual needs to be more aggressive so that it can win a greater share than its opponent, since “hurting” its opponent leads directly to improving its proportion in equation (27).

### How the Expected Payoffs and the Division of Resources Change When Varying *V* and *C*

**Fig. 3:**
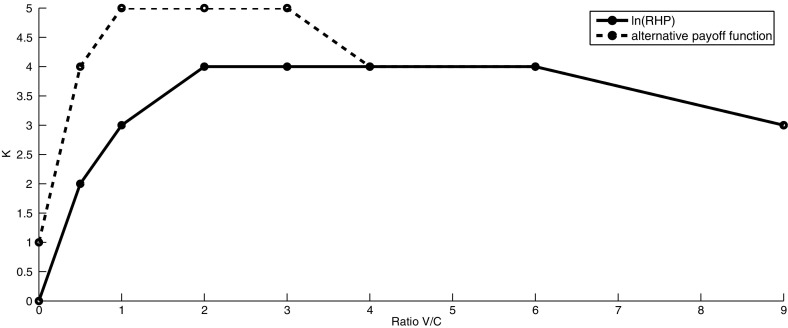
The evolutionarily stable strategy *k* for variable *V* and fixed *C* ($C=0.1$) for $\ln ({\hbox {RHP}})$ and alternative payoff function. When $C=0$, the ESS will be the highest possible value of *k* ($C\rightarrow 0 \Longrightarrow k\rightarrow \infty$)

**Fig. 4:**
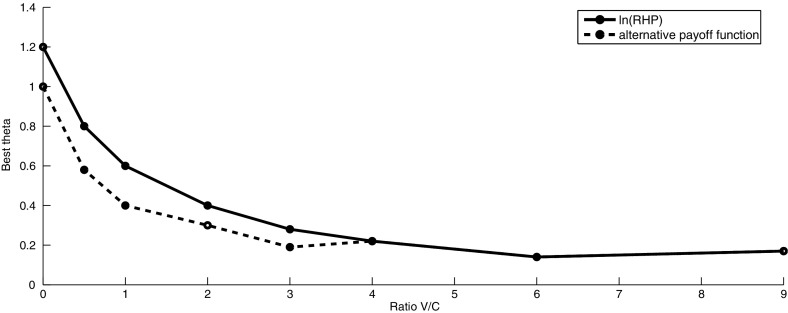
The evolutionarily stable strategy $\theta$ for variable *V* and fixed *C* ($C=0.1$) for $\ln ({\hbox {RHP}})$ and alternative payoff function. When $C=0$, the ESS will be the highest possible value of *k* ($C\rightarrow 0 \Longrightarrow k\rightarrow \infty$)

In this section, we will vary the values of *V* and fix the value of *C* ($C=0.1$), noting that different combinations of *V* and *C* correspond to different values of *k* for any given value of $\theta$. For each of these combinations, we find the ESS ($\theta$ and the corresponding *k* ) when $\ln ({\hbox {RHP}})$ is considered as the payoff function and when the alternative payoff function is used. The results are summarised in Figs. 3 and 4 where we plot the ratio $\frac{V}{C}$ with $C=0.1$ on the *x*-axis and the best strategy on the *y*-axis (optimal *k* in Fig. 3 and best $\theta$ in Fig. 4).

For the case when $V=0$ and $C > 0$, we expect the ESS to be the strategy where an individual retreats immediately. This is true when $\ln ({\hbox {RHP}})$ is considered as the payoff function. When the alternative payoff function is used, we obtain $k=1 (\theta =1)$ as the ESS (for $C=0.1$). Thus, in this case it is best to fight initially to potentially reduce the RHP of the opponent, as this increases the individual’s payoff function. On the other hand for $C=0$ and $V >0$, we obtain $k\rightarrow \infty$ as the ESS. This is the expected result as since there is no cost for losing, it is best to fight until the end of the competition. When $\frac{V}{C} \le 4$, we obtain lower values of $\theta$ as an ESS for the alternative payoff function than for the payoff function given by $\ln (RHP)$. This means that when resources are scarce, individuals need to be more aggressive in order to get a high payoff. For sufficiently high $\dfrac{V}{C}$ ratio, (e.g. for $\frac{V}{C} >4$), we obtain the same value of $\theta$ as an ESS for both payoff functions. The corresponding tables showing the expected payoffs for different combinations of $k_{x}$ and $k_{y}$ when *V* and *C* vary are given in Appendix.

## The *N*-Individual Model

In Sect. 3, we demonstrated how the expected payoff can be derived analytically for the situation of two interacting individuals. Generalisations of these results to situations with more than two individuals, however, have proven to be analytically intractable. To nevertheless gain insights into the behaviour of larger groups, we develop a simulation approach which determines the ESS for *N* interacting individuals. We imagine a population of 10,000*N* individuals, which at the start of the game is divided into 10,000 groups of size *N* at random. Members within each group interact as previously described, for a total of 200 contests, and record their payoff (this correspond to steps *S1*–*S2.3* ). The individuals then produce offspring proportional to their payoff to form a new generation of 10, 000*N* individuals. This process is repeated for 10, 000 generations (this corresponds to step *S3*). The algorithm which generates our approach is defined as follows.

- *S1* Initially, the *N* individuals can choose their strategies from the range
  $$\begin{aligned} \Theta =[\theta _1,\theta _2,\ldots ,\theta _{10}]=[0.1,0.2,\ldots ,1] \end{aligned}$$
  with probability $$p(\theta =\theta _k)=\frac{1}{10}$$, $$k=1,\ldots ,10$$.
  Set $$i=0$$.
- *S2.0* Set $H=[0,0,\ldots ,0]$ (*H* has dimension 10) and $j=0$.
- *S2.1* Each of the *N* individuals chooses a strategy $\theta _{x_i},\ i=1,\ldots ,N$ according to the probability function $p(\theta =\theta _k)$.
- *S2.2* Repeat the following for $T_{{\hbox {max}}}=200$ times steps.
  Randomly choose two individuals with their strategies $\theta _{x_i}$ and $\theta _{x_j}$ out of the *N* individuals and update their RHP according to Equations (3)–(6).
- *S2.3* Update the vector *H* as follows
  $$\begin{aligned} H(10\theta _{x_i})=H(10\theta _{x_i})+\ln ({\hbox {RHP}}_{x_i,200}),\quad i=1,\ldots ,N. \end{aligned}$$
  Set $$j=j+1$$. If $$j < $$10,000 go to *S2.0* otherwise to *S3*.
- *S3* Update probability function $p(\theta =\theta _k)$ as follows
  $$\begin{aligned} p(\theta =\theta _k)=\frac{H(10\theta _k)}{\sum \limits _{k=1}^{10}H(10\theta _k)}. \end{aligned}$$
  Set $$i=i+1$$. If $$i < $$10,000 go to *S2.0* otherwise the simulation is finished.

The outcome of this algorithm is the probability vector $p(\theta =\theta _k)$, and in most cases, the probability mass will be concentrated in a single strategy $\theta _k$ which represents the ESS. When this is not the case, the mean value of the strategies at the end of the simulation (i.e. after 10,000 generations) will be considered as the ESS. In order to analyse the accuracy of the simulation algorithm, we consider the same parameter constellation as in Sect. 3.4, namely $N=2$ and $V=C=0.1$, and determine the ESS. We obtain $p(\theta =0.6)=1$ and conclude that $\theta =0.6$ is the ESS, which falls within the [0.55, 0.67] range; the result that we obtained from equation (21). We considered other values of *V* and *C* as well, and in all situations, analytical and simulation results coincided.

### Example: Population Size $N=4$

**Fig. 5:**
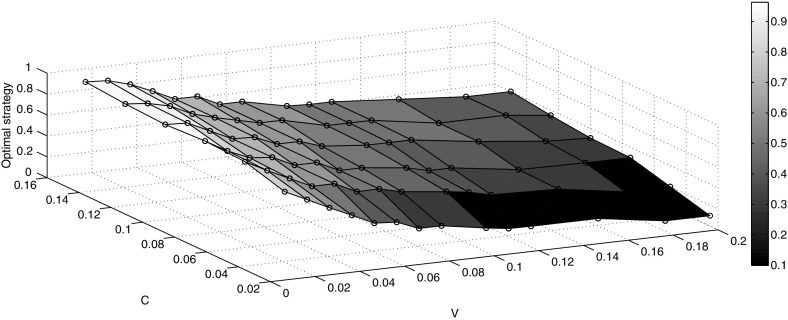
The ESS theta for different combinations of *V* and *C*

Now we consider a group of $N=4$ individuals and use the simulation algorithm described above to determine the ESSs. We do this for different combinations of *V* and *C*, and the results are shown in Table 3 and Fig. 5.

**Table 3:** The ESS value of $\theta$ for different combinations of *V* and *C*

	$C=0.025$	$C=0.05$	$C=0.075$	$C=0.1$	$C=0.125$	$C=0.15$
$V=0.01$	0.9400	0.9900	1.0000	1.0000	1.0000	1.0000
$V=0.02$	0.8800	0.9000	0.9200	0.9500	0.9700	0.9900
$V=0.03$	0.7000	0.8100	0.9000	0.9000	0.9200	0.9400
$V=0.04$	0.6000	0.8000	0.8000	0.8700	0.9000	0.9100
$V=0.05$	0.4900	0.7000	0.7900	0.8000	0.8000	0.9000
$V=0.06$	0.4000	0.6200	0.7000	0.7700	0.8000	0.8000
$V=0.07$	0.3700	0.6000	0.6900	0.7000	0.7600	0.8000
$V=0.08$	0.3000	0.5000	0.6000	0.6900	0.7000	0.7000
$V=0.09$	0.2900	0.5000	0.6000	0.6000	0.6500	0.6500
$V=0.1$	0.2600	0.4400	0.5100	0.6000	0.6000	0.6900
$V=0.11$	0.2100	0.4000	0.5000	0.5300	0.6000	0.6100
$V=0.12$	0.2000	0.4000	0.5000	0.5100	0.6000	0.6000
$V=0.15$	0.2000	0.3000	0.4000	0.5000	0.5000	0.5100
$V=0.18$	0.1400	0.2900	0.3000	0.4000	0.4300	0.4400

The ESS values show that when the value of *C* is increased for a fixed value of *V*, the value of $\theta$ is also increased. This means that the individuals fight less as the cost of injury, for example, is increased. On the other hand, when *V* is increased for a fixed *C*, we notice that the value of $\theta$ is decreased, and thus, individuals are fighting longer. If $V=C$, then the value of the ESS decreases when *V* and *C* are simultaneously increased by the same factor. This is supported by the results of $V=C=0.05$, $V=C=0.1$ and $V=C=0.15$ which have respective ESSs 0.6, 0.49 and 0.45.

Next we compare the ESSs when we increase the group size from 2 to 4 individuals. In Table 4, we show the values of the ESS for these two group sizes for some combinations of *V* and *C*. We conclude that as the group size is increased the values of strategies $\theta$ are also increased. This implies less aggressiveness in larger groups. Hence, in larger group sizes it is best to fight less than it is in smaller populations, because an individual will suffer a larger loss in RHP for fighting longer and potentially losing against three individuals.

**Table 4:** The ESS values for different combinations of *V* and *C* for $N=2$ and $N=4$

	$N=2$	$N=4$
$V=0.1, C=0.2$	[0.53, 0.73]	0.9
$V=0,1, C=0.1$	[0.55, 0.67]	0.6
$V=0.2, C=0.1$	[0.32, 0.42]	0.5
$V=0.3, C=0.1$	[0.23, 0.33]	0.35

For $N=2$, there is a range of strategies $\theta$ that correspond to the same critical value of the excess number of defeats *k* leading to concession. This range is determined by (23)

## Comparison of Strategies

In the above sections, we have derived how the ESS for different values of *C* and *V* can be calculated. Now we explore whether the knowledge about the ESS in a specific situation characterised by *V* and *C* allows us to infer the ESS for a related situation with $\alpha V$ and $\alpha C$ (for sufficiently small $\alpha$). Similarly to the Hawk–Dove game, the ratio $\frac{V}{C}$ might be the most important aspect regarding the expected payoffs (if $V<C$ the ESS of the Hawk–Dove game is simply play Hawk with probability $p=\frac{V}{C}$), as opposed to specific values of *V* and *C*. This means that if we know the ESS for small values of *V* and *C*, we can also calculate the ESS for $\alpha V$ and $\alpha C$. The following holds

28 $$\begin{aligned}&d_{x}=\frac{-\ln (\theta _{x})}{\ln (1+V)-\ln (1-C)}\approx \frac{-\ln (\theta _{x})}{V- (-C)}=\frac{-\ln (\theta _{x})}{V+C} \Rightarrow \\&d_{x}(V+C)=-\ln (\theta _{x})\nonumber \end{aligned}$$

where $\theta _{x}$ is the strategy for individual *x*. If we multiply *V* and *C* by $\alpha$, we obtain:

29 $$\begin{aligned}&d_{x}=\frac{-\ln (\theta '_{x})}{\ln (1+\alpha V)-\ln (1-\alpha C)}\approx \frac{-\ln (\theta '_{x})}{\alpha V+\alpha C}=\frac{-\ln (\theta '_{x})}{\alpha (V+C)} \Rightarrow \\&\alpha d_{x}(V+C)=-\ln (\theta '_{x})\nonumber \end{aligned}$$

where $\theta '_{x}$ is the strategy of individual *x* when *V* and *C* become $\alpha V$ and $\alpha C$, respectively. Now from equations (28) and (29) we obtain

30 $$\begin{aligned} \ln (\theta '_{x})=\alpha \ln (\theta _{x}) \Rightarrow \nonumber \\ \theta '_{x}=\theta _{x}^{\alpha }. \end{aligned}$$

This means that if for a sequence of wins and losses individual *x* retreats following strategy $\theta _{x}$, it will retreat for the same sequence following strategy $\theta '_{x}=\theta _{x}^{\alpha }$ when *V* and *C* are exchanged for $\alpha V$ and $\alpha C$, respectively (assuming that changing the value of *V* using $\alpha$ in this way does not affect the choice of $k_{x}$). Thus, if only the ratio $\frac{V}{C}$ matters for finding the ESS and $\theta _{x}$ is the ESS for V and C, then $\theta '_{x}$ will be the ESS for $\alpha V$ and $\alpha C$. We illustrate this point with an example. We assume the parameter constellation $N=2$, $V=0.02$, $C=0.04$ and $\alpha =\frac{3}{2}$ and use the simulation algorithm given in Sect. 4 to determine the ESS. We obtain $\theta _{x}=0.91$ (corresponding to $k_{x}=2$) as the ESS for $V=0.02$, $C=0.04$ and $\theta _{x}=0.87$ (corresponding to $k_{x}=2$) for $\alpha V=0.03$ and $\alpha C=0.06$. When we use formula (30) and take $\theta _{x}=0.91$ as the ESS baseline ($V=0.02$, $C=0.04$), we obtain $\theta '_{x}=0.91^{\frac{3}{2}}=0.868$ as the new ESS which is close to the 0.87 value that we get from the simulations. Thus, the results from these simulations support formula (30). We have also analysed different values of $\alpha =2,\frac{1}{2}, \frac{1}{5}, 5$ and we obtain ESS corresponding to $k_{x}=2$ for all the cases. We can conclude that equation (30) gives a good approximation for the ESS. This is always true when we have small values of *V* and *C*; however, there are some cases when it works less well, principally where *V*, *C* (or $\alpha$ which will lead to large *V* or *C* in the comparative model) is large. We note that the larger *V* and *C*, and the bigger *T*, the more unrealistic multiplying the RHP by a constant after every contest is. On the other hand the smaller *T*, there are more times when we cannot distinguish between a pair of individuals as neither of them has retreated. Thus, a realistic model should only contain relatively small *V* and *C*.

## Discussion

In this paper, we have introduced game-theoretical elements to the winner–loser model developed in Dugatkin (Dugatkin 1997; Dugatkin and Dugatkin 2007). We considered a group of individuals that are characterised by their fighting ability score (their RHP) and a strategy $\theta$ that indicates whether an individual would engage in an aggressive interaction or retreat. All individuals were assumed to possess the same RHP initially. We have developed a model that determines the expected payoff and ESS for different group sizes and payoffs, involving *V* and *C*, in such a population.

In the first part of this paper, we derived analytical results for a group of two individuals for the expected payoff and find the ESS, using $\ln (RHP)$ as the payoff function, which correspond to situations with unlimited resources. In order to calculate the expected payoff for individual *x* with strategy $\theta _{x}$, we first found the condition when this individual would retreat, represented by *k*. The variable *k* describes the critical difference between the number of wins and losses, below which individual *x* retreats. Given that a win increases the value of RHP, the value of *k* corresponds to the difference in RHP and thus only the individuals with a high RHP relative to its opponent risk engaging in an agonistic interaction to obtain more access to the available resources. We showed that there is a range of strategies $\theta _{x}$ that correspond to the same value of *k*, meaning that they will give the same payoff. Furthermore different combinations of *V* and *C* yield different ranges of $\theta _{x}$ for any given value of *k*.

We illustrated this analytical part with an example where we assumed $V=C=0.1$. We found the expected payoff for different strategies $\theta \ge 0$. In this case, we obtained a pure ESS which was achieved for $k=3$, corresponding to the $\theta$ range [0.55, 0.67]. Any strategy from this range gives the same payoff and is an ESS. We next varied *V* and *C* and saw the effect of this variations on the expected payoff and the ESS. As expected, if *V* is increased for a fixed *C*, the individuals will fight more, corresponding to lower values of $\theta$. On the other hand, if *C* is increased for a fixed *V*, we get bigger values of $\theta$ as an ESS. This means that individuals will fight less as *C* is increased.

We also used the idea of the reproductive skew (Broom et al. 2009; Keller and Reeve 1994; Reeve and Keller 2001; Shen and Reeve 2010; Vehrencamp 1983) to study how scarce resources are divided between a pair of individuals by using an alternative payoff function given in equation (27). When comparing the results with the ones obtained for the original payoff function, we observe smaller values of $\theta$ as an ESS. This means that in this case individuals need to be more aggressive in order to obtain a larger share of the available resources.

While in our model, and in those of Dugatkin (1997) and Dugatkin and Dugatkin (2007), linear hierarchies are generally formed efficiently when (i) winner and loser effects are both present, (ii) only the winner effect or (iii) only the loser effect is present, the three models give clearly distinct predictions. With only the winner effect present, individuals in our model (for optimal strategy choice) and that of Dugatkin (1997) will continue fighting indefinitely, whereas in Dugatkin and Dugatkin (2007) individuals start fighting, but eventually contests cease. With only the loser effect present, individuals would give up immediately in our model (at least for the plentiful resources case defined by payoff function (11)), would give up after the first loss in the model of Dugatkin (1997), and would fight for some longer period in the model of Dugatkin and Dugatkin (2007). These differences in the results of the three models are rooted in the modelling assumptions. In Dugatkin (1997), there is no strategic choice and individuals do not know their opponent’s RHP; in Dugatkin and Dugatkin (2007), there is no strategic choice, but they do know their opponent’s RHP, and in our model, there is strategic choice and their opponent’s RHP is known. Thus, Dugatkin and Dugatkin (2007) can be thought of as an intermediate model between the other two. However, the predictions of our model are closer to that of Dugatkin (1997) than Dugatkin and Dugatkin (2007) and we would argue that these are more realistic.

Other authors have considered alternative game-theoretical models of dominance hierarchy formation. A good recent survey which raises some interesting questions and suggestions for further modelling is Mesterton-Gibbons et al. (2016). We shall discuss two such models. Van Doorn and co-workers Doorn et al. (2003) analysed the evolution of dominance hierarchies by assuming that individuals are identical in ability throughout the time of their interaction, and so while their strategic choices depend upon past results, the actual probability of winning a contest depends upon the strategic choices of individuals, rather than their actual abilities. This is an example of what Maynard Smith Maynard Smith (1982) called an uncorrelated asymmetry (as opposed to a correlated asymmetry, as in our model). They found several evolutionary equilibria, one of them was the “dominance” equilibrium with the winner and loser effect where previous winners were more likely to take part in aggressive interactions and previous losers less likely to be aggressive. He also found a paradoxical equilibria where the higher position was occupied by the loser of an aggressive interaction than the winner. These results are very similar to the owner–intruder game Maynard Smith (1982) where paradoxical convention-based outcomes can occur. They then extended this model to larger group sizes Doorn et al. (2003), where the individuals still had limited information about previous fights. Similar as in the two-player model, several evolutionary equilibria were found, one being with the winner and loser effect. The assumptions and outcomes are thus rather different to our model.

Fawcett and Johnstone (2010) developed a model to analyse the level of aggression where each individual differed in strength, but where they had no information about this difference. They predicted that the level of aggression is related to the amount of information that an individual has about prior contests. While the young individuals should be more aggressive as they are not sure about their fighting ability, the older one are not. They have knowledge of prior experience, and they retreat after a series of losses. Although the mechanisms differ, the actual way that the populations evolve is quite similar to ours. In their model, there are real differences between individuals, but the individuals start with no knowledge and learn over time; in our model, individuals have varying probabilities of being able to win a contest, which change (perhaps due to psychological factors) over time. In each case, after a time it is clear which individuals are the better ones, and the level of aggressive interactions declines, as more individuals play the more passive strategy. We note that in their model, the eventual division into mainly aggressive strong individuals and mainly passive weak individuals is dependent upon an intermediate number of strong/ weak individuals and that this divide would not happen for all population divisions.

In each of the strategic models discussed above (Fawcett and Johnstone 2010; Doorn et al. 2003 and Doorn et al. 2003, in addition to ours), individuals face a potentially long sequence of contests where they have two options at each step. Thus, in the same way as in games such as the classical iterated prisoner’s dilemma Axelrod (1984), there is a vast array of potential strategies. Each model reduces the dimensions of this strategy space in different ways . In the models of Doorn et al. (2003), Doorn et al. (2003), individuals were constrained to have a memory only of the latest interaction with an individual and so could base their play only on the results of this latest interaction (from the iterated prisoner’s dilemma “tit for tat” is such a strategy). Fawcett and Johnstone (2010) allow individuals to know their performance from all past contests, but allow them only to condition play on the total number of contests encountered, together with the number of wins in these contests. Our model behaves in a similar way to that of Fawcett and Johnstone (2010), basing strategy on the RHP, which in turn depends directly upon the number of won and lost contests of the participating individuals.

Similar results to those from our model concerning aggression levels have been found in experimental settings. Kotrschal et al. Kotrschal et al. (1993) performed a feeding experiment with greylag geese. Grained food was given in high, medium and low density. The geese were fed twice daily, and the level of aggression was recorded. They found a low number of agonistic interactions in the high food density setting and an increase in those aggressive interactions when the food density was decreased. Nie et al. Nie et al. (2013) conducted feeding experiments with varying levels of predation with root voles. They considered four treatments by combining different levels of predation and food supply (i.e. (no predation, food), (predation, food), (predation, no food), (no predation, no food)). They observed higher levels of aggressiveness in the groups treated with unfavourable conditions such as (predation, no food) compared to groups treated with (no predation, food). When the groups were treated with (predation, food) and (no predation, no food), the level of aggression observed was intermediate. These findings support our results that if resources are scarce, then an individual needs to be more aggressive.

An important concept related to the expected payoff is that of the stopping time. The stopping time is defined as the first time when one of the two individuals hits its stopping value of *k*. It gives a guideline for how many agonistic interactions we need to observe in a pair of individuals before one retreats. After hitting the stopping time, an individual would then always retreat afterwards. We showed in our example that twenty possible interactions is enough for an individual to retreat in almost all cases. Note that if $T_{max}$ is relatively larger than the stopping time, the continued increase in the winner’s RHP after the stopping time is unrealistic. If, however, $T_{max}$ is smaller than the stopping time, it is more difficult to distinguish between a pair of individuals in terms of their ranks in the hierarchy.

Analytical results can be derived for a group of two individuals, but for larger group sizes those derivations become effectively intractable. To explore the behaviour of larger group sizes, in particular to find the ESS, we developed in the second part of the paper a simulation approach. Analysing a group of four individuals, we found that the value of the ESS is increased when *V* is increased (for a fixed *C*), and by contrast, the value of the ESS is decreased when *C* is increased (for a fixed *V*). Comparing the values of ESS for a group of two individuals with the ones obtained for a group of four individuals leads to the conclusion that individuals should be less aggressive (i.e. fight less) in larger groups.

While this result is commonly observed in behavioural experiments, there are experimental settings leading to contradictory conclusions. For example, Nicol et al. Nicol et al. (1999) conducted a feeding experiment with Isa brown birds. They analysed the behaviour of the birds in groups of four different sizes (72, 168, 264 and 368). The birds were fed twice a day, and the number of aggressive pecking interactions were recorded. The results suggested a higher level of aggression in the smallest group (72) compared to the larger groups (168, 264, 368). Further, Anderson et al. Andersen et al. (2004) compared their model predictions (larger group sizes result in lower aggression levels) with results from an experiment with crossbred pigs. They considered three groups of 6, 12 and 24 pigs (which had not interacted with each other previously) which were put into pens and the space per individual was kept the same. There was one feeder per six pigs, and they were fed on ‘Format Start’ every morning. The aggressive interactions in each group were then recorded. It was observed that the level of aggression decreased with increasing group size. This result was also supported by further experiments Estevez et al. (2007), Estévez et al. (1997), Syarifuddin and Kramer (1996) Turner et al. (2001). However, Bilvci et al. Bilčık and Keeling (2000) observed the aggressive behaviour in a feeding experiment with groups of 15, 30, 60 and 120 Hisex white hens and noticed higher level of aggression in larger groups of birds than in the smaller ones.

Summarising, we presented a game-theoretical model which determines the evolutionarily stable aggression level in a populations of *N* individuals and different payoff functions, involving *V* and *C*, within a winner–loser framework. Within a group, we found that the population evolves to a unique aggression threshold, indicating that relative to their strength, all individuals adopt the same decision rule against whom to fight. Typically, the hierarchy is established quickly, with aggressive fights happening only in the early contests. Applied to real-world situations, this points to the crucial importance of the first few fights for hierarchy formation. Later fights only determine the position of lower-ranked individuals. While higher values of *C* for losing an aggressive interaction (keeping the value of *V* constant) lead to lower aggression levels in the population, the reverse is true for increasing the value *V* for winning an aggressive interaction (keeping *C* constant): the higher the value of *V*, the higher is the aggression level in the population. Further, we predict lower aggression levels in larger populations. Our results are largely supported by experimental evidence so that we conclude that the introduction of game-theoretical elements to winner–loser models provides a further step towards a realistic description of aggressive interactions.

## Appendix: Expected Payoffs for Different *V* and *C*

In this section we show the expected payoffs for different combinations of $k_{x}$ and $k_{y}$ where both the $\ln (RHP)$ and alternative payoff function are considered as payoffs. See Tables (5, 6, 7, 8, 9, 10, 11, 12, 13, and 14).

**Table 5:** Expected payoffs for different values of *k* when $V=0.1, C=0$

	$k_{y}=1$($\theta _{y}=1$)	$k_{y}=2$ ($\theta _{y}=0.9$)	$k_{y}=3$ ($\theta _{y}=0.8$)	$k_{y}=4$ ($\theta _{y}=0.7$)	$k_{y}=5$ ($\theta _{y}=0.65$)	$k_{y}=6$ ($\theta _{y}=0.6$)	$k_{y}=25$ ($\theta _{y}=0.1)$
$k_{x}=1$ ($\theta _{x}=1$)	3.2560	2.8414	2.8414	2.7754	2.7392	2.7131	2.6645
$k_{x}=2$ ($\theta _{x}=0.9$)	3.5456	3.2550	3.1092	3.0271	2.9705	2.9417	2.8767
$k_{x}=3$ ($\theta _{x}=0.8$)	3.6699	3.4022	3.2517	3.1682	3.1168	3.0786	3.0138
$k_{x}=4$ ($\theta _{x}=0.7$)	3.7360	3.4843	3.3432	3.2572	3.1991	3.1643	3.0995
$k_{x}=5$ ($\theta _{x}=0.65$)	3.7721	3.5409	3.3946	3.3123	3.2588	3.2175	3.1514
$k_{x}=6$ ($\theta _{x}=0.6$)	3.7983	3.5697	3.4328	3.3471	3.2939	3.2570	3.1855
$k_{x}=25$ ($\theta _{x}=0.1$)	3.8469	3.6347	3.4976	3.4119	3.3600	3.3259	3.2551

**Table 6:** Division of resources for different values of *k* when $V=0.1, C=0$

	$k_{y}=1$ ($\theta _{y}=1$)	$k_{y}=2$ ($\theta _{y}=0.9$)	$k_{y}=3$ ($\theta _{y}=0.8$)	$k_{y}=4$ ($\theta _{y}=0.7$)	$k_{y}=5$ ($\theta _{y}=0.65$)	$k_{y}=6$ ($\theta _{y}=0.6$)	$k_{y}=25$ ($\theta _{y}=0.1$)
$k_{x}=1$ ($\theta _{x}=1$)	0.500	0.4555	0.4364	0.4262	0.4207	0.4167	0.4092
$k_{x}=2$ ($\theta _{x}=0.9$)	0.5445	0.500	0.4775	0.4649	0.4562	0.4518	0.4418
$k_{x}=3$ ($\theta _{x}=0.8$)	0.5636	0.5225	0.4994	0.4866	0.4787	0.4728	0.4629
$k_{x}=4$ ($\theta _{x}=0.7$)	0.5738	0.5351	0.5134	0.500	0.4913	0.4860	0.4760
$k_{x}=5$ ($\theta _{x}=0.65$)	0.5793	0.5438	0.5213	0.5087	0.500	0.4941	0.4840
$k_{x}=6$ ($\theta _{x}=0.6$)	0.5833	0.5482	0.5272	0.5140	0.5059	0.500	0.4892
$k_{x}=25$ ($\theta _{x}=0.1$)	0.5908	0.5582	0.5371	0.5240	0.5160	0.5108	0.5000

**Table 7:** Expected payoffs for different values of *k* when $V=0, C=0.1$

	$k_{y}=0$ ($\theta _{y}=1.2$)	$k_{y}=1$ ($\theta _{y}=1$)	$k_{y}=2$ ($\theta _{y}=0.9$)	$k_{y}=3$ ($\theta _{y}=0.8$)	$k_{y}=4$ ($\theta _{y}=0.7$)	$k_{y}=5$ ($\theta _{y}=0.6$)	$k_{y}=22$ ($\theta _{y}=0.1$)
$k_{x}=0$ ($\theta _{x}=1.2$)	2.3026	2.3026	2.3026	2.3026	2.3026	2.3026	2.3026
$k_{x}=1$ ($\theta _{x}=1$)	2.3026	2.2500	2.2122	2.1559	2.1156	2.0843	2.0052
$k_{x}=2$ ($\theta _{x}=0.9$)	2.3026	2.2099	2.1723	2.0648	2.0038	1.9601	1.8634
$k_{x}=3$ ($\theta _{x}=0.8$)	2.3026	2.1452	2.0539	1.9020	1.8218	1.7661	1.6553
$k_{x}=4$ ($\theta _{x}=0.7$)	2.3026	2.0961	1.9798	1.8064	1.7194	1.6621	1.5486
$k_{x}=5$ ($\theta _{x}=0.6$)	2.3026	2.0508	1.9208	1.7315	1.6421	1.5854	1.4706
$k_{x}=22$ ($\theta _{x}=0.1$)	2.3026	1.8927	1.7268	1.5113	1.4190	1.3606	1.2500

**Table 8:** Division of resources for different values of *k* when $V=0, C=0.1$

	$k_{y}=0$ ($\theta _{y}=1.2$)	$k_{y}=1$ ($\theta _{y}=1$)	$k_{y}=2$ ($\theta _{y}=0.9$)	$k_{y}=3$ ($\theta _{y}=0.8$)	$k_{y}=4$ ($\theta _{y}=0.7$)	$k_{y}=5$ ($\theta _{y}=0.6$)	$k_{y}=22$ ($\theta _{y}=0.1$)
$k_{x}=0$ ($\theta _{x}$=1.2)	0.500	0.500	0.500	0.500	0.500	0.500	0.500
$k_{x}=1$ ($\theta _{x}=1$)	0.500	0.500	0.5004	0.5019	0.5038	0.5068	0.5293
$k_{x}=2$ ($\theta _{x}=0.9)$	0.500	0.4996	0.500	0.5020	0.5049	0.5084	0.5362
$k=3$ $(\theta _{x}=0.8)$	0.500	0.4981	0.4980	0.500	0.5035	0.5081	0.5395
$k_{x}=4$ ($\theta _{x}=0.7$)	0.500	0.4962	0.4951	0.4965	0.500	0.5048	0.5364
$k_{x}=5$ ($\theta _{x}=0.6$)	0.500	0.4932	0.4916	0.4919	0.4952	0.500	0.5317
$k_{x}=22$ ($\theta _{x}=0.1$)	0.500	0.4707	0.4638	0.4605	0.4636	0.4683	0.5000

**Table 9:** Expected payoffs for different values of *k* when $V=0.2, C=0.1$

	$k_{y}=0$ ($\theta _{y}=1.2$)	$k_{y}=1$ ($\theta _{y}=0.9$)	$k_{y}=2$ ($\theta _{y}=0.7$)	$k_{y}=3$ ($\theta _{y}=0.5$)	$k_{y}=4$ ($\theta _{y}=0.4$)	$k_{y}=5$ ($\theta _{y}=0.3$)	$k_{y}=9$ ($\theta _{y}=0.1$)
$k_{x}=0$ ($\theta _{x}=1.2$)	2.3026	2.3026	2.3026	2.3026	2.3026	2.3026	2.3026
$k_{x}=1$ ($\theta _{x}=0.9$)	5.9490	4.0724	3.5544	3.3444	3.2392	3.2011	3.1203
$k_{x}=2$ ($\theta _{x}=0.7$)	5.9490	4.4956	3.9443	3.6854	3.5594	3.4934	3.4014
$k_{x}=3$ ($\theta _{x}=0.5$)	5.9490	4.6207	4.0597	3.7925	3.6494	3.5800	3.4715
$k_{x}=4$ ($\theta _{x}=0.4$)	5.9490	4.6521	4.0743	3.8035	3.6629	3.5895	3.4778
$k_{x}=5$ ($\theta _{x}=0.3$)	5.9490	4.6299	4.0573	3.7795	3.6393	3.5616	3.4574
$k_{x}=9$ ($\theta _{x}=0.1$)	5.9490	4.5454	3.9244	3.6488	3.5102	3.4255	3.3109

**Table 10:** Division of resources for different values of *k* when $V=0.2, C=0.1$

	$k_{y}=0$ ($\theta _{y}=1.2$)	$k_{y}=1$ ($\theta _{y}=0.94$)	$k_{y}=2$ ($\theta _{y}=0.7$)	$k_{y}=3$ ($\theta _{y}=0.5$)	$k_{y}=4$ ($\theta _{y}=0.4$)	$k_{y}=5$ ($\theta _{y}=0.3$)	$k_{y}=9$ ($\theta _{y}=0.1$)
$k_{x}=0$ ($\theta _{x}=1.2$)	0.500	0.2790	0.2790	0.2790	0.2790	0.2790	0.2790
$k_{x}=1$ ($\theta _{x}=0.9$)	0.7210	0.500	0.4430	0.4233	0.4161	0.4161	0.4227
$k_{x}=2$ ($\theta _{x}=0.7$)	0.7210	0.5570	0.500	0.4781	0.4712	0.4701	0.4805
$k_{x}=3$ ($\theta _{x}=0.5$)	0.7210	0.5767	0.5219	0.500	0.4923	0.4916	0.5012
$k_{x}=4$ ($\theta _{x}=0.4$)	0.7210	0.5839	0.5288	0.5077	0.500	0.4991	0.5084
$k_{x}=5$ ($\theta _{x}=0.3$)	0.7210	0.5834	0.5299	0.5084	0.5009	0.500	0.5104
$k_{x}=9$ ($\theta _{x}=0.1$)	0.7210	0.5773	0.5195	0.4988	0.4916	0.4896	0.5000

**Table 11:** Expected payoffs for different values of *k* when $V=0.1, C=0.2$

	$k_{y}=0$ ($\theta _{y}=1.2$)	$k_{y}=1$ ($\theta _{y}=1$)	$k_{y}=2$ ($\theta _{y}=0.70$)	$k_{y}=3$ ($\theta _{y}=0.50$)	$k_{y}=4$ ($\theta _{y}=0.30$)	$k_{y}=5$ ($\theta _{y}=0.25$)	$k_{y}=8$ ($\theta _{y}=0.10$)
$k_{x}=0$ ($\theta _{x}=1.2$)	2.3026	2.3026	2.3026	2.3026	2.3026	2.3026	2.3026
$k_{x}=1$ ($\theta _{x}=1$)	4.2088	3.1454	2.8027	2.6514	2.5755	2.5237	2.4678
$k_{x}=2$ ($\theta _{x}=0.70$)	4.2088	3.2896	2.8714	2.6631	2.5416	2.4852	2.4037
$k_{x}=3$ ($\theta _{x}=0.50$)	4.2088	3.2705	2.8019	2.565	2.4378	2.3715	2.2812
$k_{x}=4$ ($\theta _{x}=0.30$)	4.2088	3.2019	2.7000	2.4429	2.3217	2.2399	2.1442
$k_{x}=5$ ($\theta _{x}=0.25$)	4.2088	3.1383	2.5824	2.3190	2.1796	2.1203	2.0265
$k_{x}=8$ ($\theta _{x}=0.10$)	4.2088	2.9289	2.2914	2.0099	1.8699	1.8030	1.7182

**Table 12:** Division of resources for different values of *k* when $V=0.1, C=0.2$

	$k_{y}=0$ ($\theta _{y}=1.2$)	$k_{y}=1$ ($\theta _{y}=1$)	$k_{y}=2$ ($\theta _{y}=0.70$)	$k_{y}=3$ ($\theta _{y}=0.50$)	$k_{y}=4$ ($\theta _{y}=0.30$)	$k_{y}=5$ ($\theta _{y}=0.25$)	$k_{y}=8$ ($\theta _{y}=0.10$)
$k_{x}=0$ ($\theta _{x}=1.2$)	0.5000	0.3536	0.3536	0.3536	0.3536	0.3536	0.3536
$k_{x}=1$ ($\theta _{x}=1$)	0.6464	0.5000	0.4630	0.4554	0.4600	0.4679	0.5091
$k_{x}=2$ ($\theta _{x}=0.70$)	0.6464	0.5370	0.5000	0.4938	0.5006	0.5171	0.5786
$k_{x}=3$ ($\theta _{x}=0.50$)	0.6464	0.5446	0.5062	0.5000	0.5092	0.5268	0.5936
$k_{x}=4$ ($\theta _{x}=0.30)$	0.6464	0.5400	0.4994	0.4905	0.5000	0.5177	0.5860
$k_{x}=5$ ($\theta _{x}=0.25)$	0.6464	0.5321	0.4829	0.4732	0.4823	0.5000	0.5684
$k_{x}=8$ ($\theta _{x}=0.10)$	0.6464	0.4909	0.4214	0.4064	0.4140	0.4316	0.5000

**Table 13:** Expected payoffs for different values of *k* when $V=0.3, C=0.1$

	$k_{y}=0$ ($\theta _{y}=1.2$)	$k_{y}=1$ ($\theta _{y}=0.85$)	$k_{y}=2$ ($\theta _{y}=0.59$)	$k_{y}=3$ ($\theta _{y}=0.41$)	$k_{y}=4$ ($\theta _{y}=0.28$)	$k_{y}=5$ ($\theta _{y}=0.19$)	$k_{y}=6$ ($\theta _{y}=0.13$)
$k_{x}=0$ ($\theta _{x}=1.2)$	2.3026	2.3026	2.3026	2.3026	2.3026	2.3026	2.3026
$k_{x}=1$ ($\theta _{x}=0.85)$	7.5500	4.8750	4.1779	3.9227	3.8359	3.7927	3.7710
$k_{x}=2$ ($\theta _{x}=0.59)$	7.5500	5.4765	4.7500	4.4620	4.3284	4.2858	4.2275
$k_{x}=3$ ($\theta _{x}=0.41)$	7.5500	5.6512	4.9101	4.9000	4.4783	4.4089	4.3710
$k_{x}=4$ ($\theta _{x}=0.28)$	7.5500	5.6705	4.9720	4.6375	4.5023	4.4298	4.4038
$k_{x}=5$ ($\theta _{x}=0.19)$	7.5500	5.6573	4.9114	4.6229	4.4937	4.4200	4.3725
$k_{x}=6$ ($\theta _{x}=0.13)$	7.5500	5.6304	4.9107	4.5955	4.4477	4.3944	4.3500

**Table 14:** Division of resources for different values of *k* when $V=0.3, C=0.1$

	$k_{y}=0$ ($\theta _{y}=1.2$)	$k_{y}=1$ ($\theta _{y}=0.85$)	$k_{y}=2$ ($\theta _{y}=0.59$)	$k_{y}=3$ ($\theta _{y}=0.41$)	$k_{y}=4$ ($\theta _{y}=0.28$)	$k_{y}=5$ ($\theta _{y}=0.19$)	$k_{y}=6$ ($\theta _{y}=0.13$)
$k_{x}=0$ ($\theta _{x}=1.2)$	0.5000	0.2337	0.2337	0.2337	0.2337	0.2337	0.2337
$k_{x}=1$ ($\theta _{x}=0.85)$	0.7663	0.5000	0.4342	0.4131	0.4089	0.4088	0.4104
$k_{x}=2$ ($\theta _{x}=0.59)$	0.7663	0.5658	0.5000	0.4783	0.4711	0.4727	0.4714
$k_{x}=3$ ($\theta _{x}=0.41)$	0.7663	0.5869	0.5217	0.5000	0.4936	0.4926	0.4940
$k_{x}=4$ ($\theta _{x}=0.28)$	0.7663	0.5911	0.5289	0.5064	0.5000	0.4986	0.5016
$k_{x}=5$ ($\theta _{x}=0.19)$	0.7663	0.5912	0.5273	0.5074	0.5014	0.5000	0.5007
$k_{x}=6$ ($\theta _{x}=0.13)$	0.7663	0.5896	0.5286	0.5060	0.4984	0.4993	0.5000
